# Trophoblast cell-surface antigen 2 (TROP2) expression in triple-negative breast cancer

**DOI:** 10.1186/s12885-022-10076-7

**Published:** 2022-09-24

**Authors:** Yeonjin Jeon, Uiree Jo, Jongmoo Hong, Gyungyub Gong, Hee Jin Lee

**Affiliations:** 1 Department of Pathology, University of Ulsan College of Medicine, Asan Medical Center, 88 Olympic-ro 43-gil, Songpa-gu, Seoul, 138-736 Korea; 2 Biomedical Sciences, Asan Medical Institute of Convergence Science and Technology (AMIST), Asan Medical Center, University of Ulsan College of Medicine, 88 Olympic-ro 43-gil, Songpa-gu, Seoul, 138-736 Korea

**Keywords:** TROP2, Triple-negative breast cancer, Overall survival

## Abstract

**Background:**

Trophoblast cell-surface antigen 2 (TROP2) is related to tumor proliferation enhancement and poor prognosis. An antibody targeting TROP2 was developed to treat metastatic triple-negative breast cancer (TNBC) which has a limited treatment modality. To characterize the TROP2 expressing tumors in TNBC, we analyzed TROP2 expression in three cohorts; (1) primary tumor without neoadjuvant chemotherapy, (2) primary tumor with neoadjuvant chemotherapy, and (3) metastatic tumor.

**Methods:**

A total of 807 TNBC cases were evaluated for TROP2 immunohistochemical expression. We evaluated the TROP2 H-score distribution in the three cohorts. Tumors were divided into two groups based on TROP2 expression (high vs. low). We analyzed the relationship between clinicopathologic features and markers, including epidermal growth factor receptor, cytokeratin 5/6, p53, and Ki-67, and prognostic significance at high vs. low TROP2 expression.

**Results:**

There was no difference in TROP2 H-score distribution between the three cohorts. Moderate-to-strong membranous expression of TROP2 in at least 10% of tumor cells was present in 662 cases (82.0%) in Cohort 1, 59 cases (89.4%) in Cohort 2, and 23 cases (88.5%) in Cohort 3. There was no significant difference in clinicopathologic features between high vs. low TROP2 in all cohorts. TROP2 H-score was an independent poor prognostic factor for overall survival in Cohort 3.

**Conclusions:**

TNBC showed similar TROP2 expression regardless of neoadjuvant treatment or primary tumor/metastasis. Although the prognostic significance of TROP2 expression in metastatic TNBC has been revealed, further evaluation of the predictive value of TROP2 expression for targeted therapy is needed.

**Supplementary Information:**

The online version contains supplementary material available at 10.1186/s12885-022-10076-7.

## Background

Breast cancer in women had the highest incidence rate (24.5%) and mortality rate (15.5%) of all cancers in 2020 [1]. Triple-negative cancer (TNBC) accounts for about 10–15% of all diagnosed breast cancer. This cancer lacks immunohistochemical expression of the estrogen receptor (ER) and progesterone receptor (PR) and does not overexpress human epidermal growth factor receptor 2 (HER2) immunohistochemically or in situ hybridization [2, 3]. TNBC tends to have larger tumors and more nodal metastasis than other subtypes. It has a bad prognosis with rapid distant recurrence [4]. The 5-year relative survival rate of TNBC is 77%, compared with 93% in other breast cancer subtypes [5]. TNBC is a heterogeneous disease that can be divided into four subtypes (luminal androgen receptor, basal-like immunosuppressed, basal-like immune-activated, and mesenchymal) by gene expression profile by Burstein et al. [6]. Because TNBC is negative for ER and HER2, it does not respond well to endocrine therapy and molecular targeted therapy, respectively. Chemotherapy is still the standard systemic treatment [7]. Especially in the case of metastatic TNBC, the median breast cancer-specific survival is only 12 months. New therapeutic agents are urgently needed [8].

Trophoblast cell-surface antigen 2 (TROP2) is one of the numerous targetable gene mutations or marker proteins studied in cancer therapy. TROP2 was discovered first in trophoblast cells as a surface marker. Gene *TACSTD2* located on chromosome 1p32 encodes TROP2 [9, 10]. TROP2 consists of extracellular and transmembrane domains and a cytoplasmic tail [11]. Mitogen-activated protein kinase (MAPK) pathway is activated by TROP2 expression. Activated MAPK pathway leads to cancer cell proliferation, invasion, migration, and survival of cancer cells [12]. TROP2 is overexpressed in several carcinomas, such as colorectal, pancreatic, gastric, oral squamous cell carcinoma, ovarian, and breast cancers, compared with the corresponding normal tissue [13–18]. In these studies, carcinomas with high TROP2 expression showed poor prognosis. Sacituzumab govitecan which is an antibody–drug conjugate (ADC) consists of an anti-TROP2 antibody and a cytotoxic drug, SN-38. It eradicates TNBC in vitro and in vivo [19]. In a clinical trial of 108 metastatic TNBC patients who had received more than one treatment and Sacituzumab govitecan for metastatic TNBC, 33.3% of patients showed complete response or partial response [20]. Based on this result, sacituzumab govitecan-hziy (Trodelvy™) received standard approval in April 2021 for the treatment of metastatic TNBC in patients who have received more than one previous therapy. In previous research, 88% of the tumors showed moderate to strong TROP2 expression [21]. Although high TROP2 expression had association with improved progression-free survival, there was no control group and the sample size was small (*n* = 48). Confirmation of high TROP2 expression is not necessary for drug usage.

We tried to determine if primary tumors with or without neoadjuvant chemotherapy and metastatic tumors in TNBC have different TROP2 expression levels. We also examined the correlation between TROP2 expression and clinicopathologic features and conducted a survival analysis.

## Materials and methods

### Patients and clinical data

This study was done in three different TNBC cohorts. The first cohort (Cohort 1) comprised 715 patients who underwent surgical treatment for breast cancer and did not receive neoadjuvant chemotherapy between 2004 and 2011. The second cohort (Cohort 2) comprised 66 patients who had residual tumors after neoadjuvant chemotherapy and who underwent surgical treatment for residual breast cancer between 2011 and 2012. The third cohort (Cohort 3) comprised 26 patients who had surgery for metastatic breast cancer between 2001 and 2016 (Supplementary Table 1). All patients in Cohort 3 were diagnosed with metastasis after primary breast cancer, except for one patient who was diagnosed with metastasis of lung and primary breast cancer simultaneously. All patients were recruited from Asan Medical Center, and follow-up data was obtained. Paraffin blocks were obtained from the surgical specimens of all patients. Clinicopathologic data were obtained through review of medical records, including age at diagnosis, sex, tumor location, neoadjuvant chemotherapy, and death. Pathologic features (nuclear/histologic grade, TNM staging, lymphovascular invasion (LVI), tumor-infiltrating lymphocytes (TILs), epidermal growth factor receptor (EGFR), cytokeratin 5/6 (CK5/6), p53, and Ki-67 immunostains) were noted after pathologic review of the surgical specimens. TNBC diagnosis was based on immune-negative results in ER and PR, HER2 negative results in immunostains or silver in situ hybridization tests. The TIL level was calculated as the proportion of the area occupied by mononuclear inflammatory cells within the tumor stroma. For EGFR, membranous staining in more than 10% of tumor cells was considered positive. For CK5/6, any tumor cell being positive was interpreted as a positive. For p53, the percentage of positive tumor cells was classified into four hierarchical groups. For Ki-67, the percentage of positive tumor cells was classified into three hierarchical groups. Our study protocol was approved by the Institutional Review Board of Asan Medical Center (2013–0866).

### Immunohistochemical staining and interpretation

Sections from tissue microarrays were immunostained using TROP2 rabbit monoclonal antibodies (EPR20043, Abcam, Cambridge, UK; dilution 1:2000). Immunostaining of the tumor specimens was performed using the autoimmunostainer Benchmark XT (Ventana Medical Systems, Tucson, AZ, USA) with Optiview Dab Detection Kit (Ventana Medical Systems, Tucson, AZ, USA) according to the manufacturers’ manuals and using the reagents provided in the kit.

In brief, sections of 4 μm were mounted on silanized slides and dried for 10 min at RT, followed by 20 min in an incubator at 65℃. Sections were conducted by HIER (CC1) for 32 min and incubated for 16 min with anti-TROP2 in the autoimmunostainer. Normal skin tissue was used for positive control and normal cerebral cortex was used for negative control of TROP2 immunostain (Supplementary Figure 1) [22, 23]. Immunostained slides were scanned on a PANNORAMIC 250 Flash III (3DHISTECH, Budapest, Hungary) with PANNORAMIC Scanner Software (3DHISTECH, Budapest, Hungary). TROP2 immunoexpression was evaluated in cells showing membranous expression. The score was obtained by using the semi-quantitative H-score method [24]. The staining intensity was as followed (0, no; 1 + , weak; 2 + , moderate; and 3 + , strong). The percentage of tumor cells showing expression was multiplied by each intensity group. The final scores were calculated by summing the values of each group. The mean TROP2 expression in the entire cohort (167) was used to discriminate between the TROP2 high group and low group.

### Statistical analysis

All statistical analysis was conducted using SPSS 20.0 statistical software (SPSS Inc, Chicago, IL, USA). The Mann–Whitney U test was performed to compare the difference in TROP2 expression between two independent cohorts. The correlations between TROP2 expression and clinicopathologic features were analyzed using the χ2 test. Log-rank tests and Kaplan–Meier curves were used for comparing survival differences between the TROP2 high and low groups. Overall time was calculated from the date of primary tumor surgery to the date of death in Cohort 1 and Cohort 2. In Cohort 3, overall time was defined as duration calculated from the date of the metastatic lesion surgery to the date of death. Univariate regression analysis using the Cox proportional hazards model was applied for estimating the hazard ratios (HRs) of the clinicopathologic features and TROP2 expression. *P* < 0.05 was considered statistically significant.

## Results

### Clinicopathologic features and TROP2 immunoexpression in three TNBC cohorts

In Cohort 1, 714 women and one man ranged in age from 23–76 (median 47) years. In Cohort 2, 66 women ranged in age from 23–70 (median 40) years. In Cohort 3, 26 women ranged in age from 25–70 (median 48) years. In Cohort 1 and Cohort 2, all patients received a breast-conserving operation or mastectomy. In Cohort 3, all patients had surgical treatment of the metastatic site. In Cohort 1, TNBC histologic type was mostly invasive breast carcinoma of no special type (IBC-NST) (83.4%), followed by metaplastic carcinoma, and carcinoma with apocrine differentiation (9.2%, and 3.6%, respectively) based on the WHO classification of breast tumors, 5^th^ edition. In Cohort 2, IBC-NST was 92.4% of TNBC, while the rest was metaplastic carcinoma (7.6%). In Cohort 3, all the TNBC was IBC-NST. The follow-up period ranged from 9–187 (median 128) months in Cohort 1, 12–102 (median 84) months in Cohort 2, and 5–113 (median 26) months in Cohort 3.

**Fig. 1:**
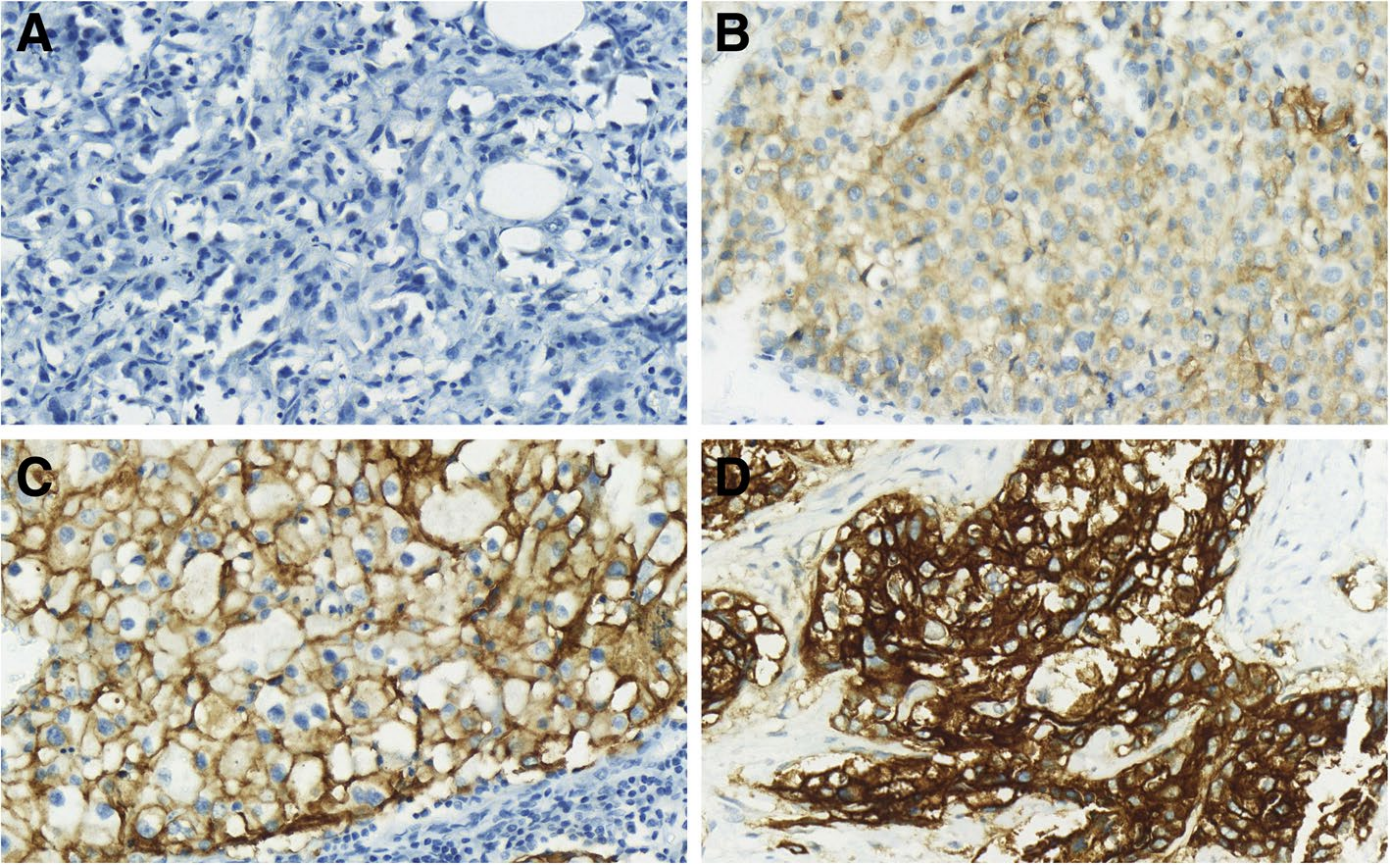
Representative TROP2 immunoexpression in TNBC. Original magnification × 200. **a** 0, no **b** 1 + , weak **c** 2 + , moderate **d** 3 + , strong

**Fig. 2:**
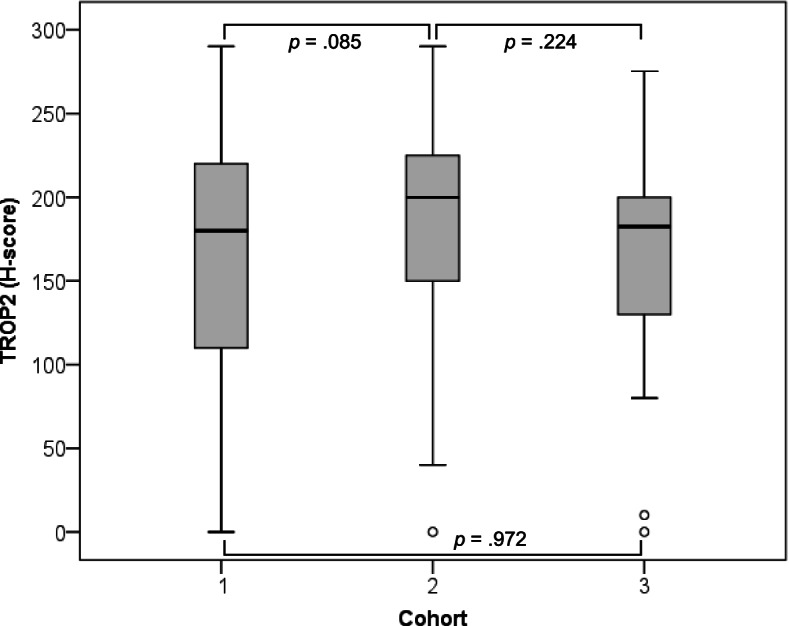
Box plots of TROP2 expression in each cohort. There was no significant difference between Cohort 1 (median [25%, 75%]; 180 [110, 220]), Cohort 2 (200 [150, 226]), and Cohort 3 (182 [130, 205]) in the Mann–Whitney U test

TROP2 expression was identified in the cellular membrane and cytoplasm of tumor cells. In previous studies, membranous TROP2 expression was related to an unfavorable prognosis in breast cancer. Membranous expression is relevant for the use of the ADC [15, 25]. Therefore, TROP2 expression was evaluated for membranous expression only, not for cytoplasmic expression (Fig. 1). The median [1^st^ quartile, 3^rd^ quartile] H-score was 180 [110, 220], 200 [150, 226], and 182 [130, 205] in Cohort 1, Cohort 2, and Cohort 3, respectively. A Mann–Whitney U test showed that H-score was not significantly different in the three cohorts (Fig. 2). Considering TROP2 expression in more than 10% of tumor cells is positive, moderate-to-strong intensity of TROP2 expression occurred in 662 cases (82.0%) in Cohort 1, 59 cases (89.4%) in Cohort 2, and 23 cases (88.5%) in Cohort 3 (Table 1a). When the expression was divided into positive or negative by 10% of any TROP2 expression intensity, positive staining was evident in 694 cases (97.1%) in Cohort 1, 65 cases (98.5%) in Cohort 2, and 24 cases (92.3%) in Cohort 3 (Table 1b).

**Table 1:** Distribution of TROP2 expression in three different cohorts according to A) dominant intensity and B) division by criteria of 10%

	Total (%)	Cohort 1 (%)	Cohort 2 (%)	Cohort 3 (%)	*p*-value
**A**					
No/Weak	151 (18.7)	140 (19.6)	7 (10.6)	4 (15.4)	.183
Moderate/Strong	656 (81.3)	575 (80.4)	59 (89.4)	22 (84.6)	
**B**					
Negative	24 (3)	21 (2.9)	1 (1.5)	2 (7.7)	.287
Positive	783 (97)	694 (97.1)	65 (98.5)	24 (92.3)	

The clinicopathologic features for the three cohorts are summarized in Table 2. TROP2 expression was not significantly associated with age, histologic subtype, nuclear and histologic grades, stage, LVI, or TIL levels.

**Table 2:** Correlation between clinicopathologic features and TROP2 expression in TNBC patients

Factors	Total (%)	TROP2 expression		*p*-value
**Cohort 1**		Low (%)	High (%)	
Age (yr)				.107
< 50	426 (59.6)	176 (56.2)	250 (62.2)	
≥ 50	289 (40.4)	137 (43.8)	152 (37.8)	
Nuclear grade				.199
1–2	184 (25.7)	88 (28.1)	96 (23.9)	
3	531 (74.3)	225 (71.9)	306 (76.1)	
Histologic grade				.477
1–2	178 (24.9)	82 (26.2)	96 (23.9)	
3	537 (75.1)	231 (73.8)	306 (76.1)	
Histologic type				.825
IBC-NST	596 (83.4)	262 (83.7)	34 (83.1)	
Non-IBC-NST	119 (16.6)	51 (16.3)	68 (16.9)	
pT				.578
1–2	689 (96.4)	303 (96.8)	386 (96.0)	
3–4	26 (3.6)	10 (3.2)	16 (4.0)	
LN status				.945
Negative	467 (65.3)	204 (65.2)	263 (65.4)	
Positive	248 (34.7)	109 (34.8)	139 (34.6)	
LVI				.989
Negative	537 (75.1)	235 (75.1)	302 (75.1)	
Positive	178 (24.9)	78 (24.9)	100 (24.9)	
Stage				.936
1	241 (33.7)	105 (33.5)	136 (33.8)	
2–3	474 (66.3)	208 (66.5)	266 (66.2)	
TILs (%)				.638
< 10	169 (23.6)	79 (25.2)	90 (22.4)	
10–59	320 (44.8)	139 (44.4)	181 (45.0)	
60–100	226 (31.6)	95 (30.4)	131 (32.6)	
**Cohort 2**		Low (%)	High (%)	
Age (yr)				.439
< 50	51 (77.3)	15 (71.4)	36 (80.0)	
≥ 50	15 (22.7)	6 (28.6)	9 (20.0)	
Nuclear grade				.955
2	16 (24.2)	5 (23.8)	11 (24.4)	
3	50 (75.8)	16 (76.2)	34 (75.6)	
Histologic grade				.979
2	19 (28.8)	6 (28.6)	13 (28.9)	
3	47 (71.2)	15 (71.4)	32 (71.1)	
Histologic type				.683
IBC-NST	61 (92.4)	19 (90.5)	42 (93.3)	
Non-IBC-NST	5 (7.6)	2 (9.5)	3 (6.7)	
ypT				.637
1–2	57 (87.7)	19 (90.5)	38 (86.4)	
3–4	8 (12.3)	2 (9.5)	6 (13.6)	
LN status				.883
Negative	40 (60.6)	13 (61.9)	27 (60.0)	
Positive	26 (39.4)	8 (38.1)	26 (39.4)	
LVI				.714
Negative	46 (69.7)	14 (66.7)	32 (71.1)	
Positive	20 (30.3)	7 (33.3)	13 (28.9)	
Stage				.547
1	31 (47.0)	11 (52.4)	20 (44.4)	
2–3	35 (53.0)	10 (47.6)	25 (55.6)	
TILs (%)				.811
< 10	35 (53.0)	10 (47.6)	25 (55.6)	
10–59	26 (39.4)	9 (42.9)	17 (37.8)	
60–100	5 (7.6)	2 (9.5)	3 (6.7)	
RCB class				.110
1	2 (3.0)	2 (9.5)	0	
2	44 (66.7)	13 (61.9)	31 (68.9)	
3	20 (30.3)	6 (28.6)	14 (31.1)	
Miller-Payne grade				.660
1	2 (3.0)	1 (4.8)	1 (2.2)	
2	12 (18.2)	3 (14.3)	9 (20.0)	
3	32 (48.5)	10 (47.6)	22 (48.9)	
4	20 (30.3)	7 (33.3)	13 (28.9)	
**Cohort 3**		Low (%)	High (%)	
Age (yr)				.837
< 50	18 (69.2)	6 (66.7)	12 (70.6)	
≥ 50	8 (30.8)	3 (33.3)	5 (29.4)	
TILs (%)				.186
< 2	7 (26.9)	1 (11.1)	6 (35.3)	
≥ 2	19 (73.1)	8 (88.9)	11 (64.7)	
Site				.299
Lung	12 (46.2)	5 (55.6)	7 (41.2)	
Brain	11 (42.3)	2 (22.2)	9 (52.9)	
Bone	2 (7.7)	1 (11.1)	1 (5.9)	
Soft tissue	1 (3.8)	1 (11.1)	0	

*IBC-NST* Invasive breast carcinoma of no special type, *pT* pathologic T stage, *LN* Lymph node, *LVI* Lymphovascular invasion, *ypT* Post neoadjuvant therapy pathologic T stage, *RCB* Residual Cancer Burden, *TILs* Tumor-infiltrating lymphocytes

### Correlation between TROP2 expression and other markers (CK5/6, EGFR, p53, and Ki-67) in Cohort 1 and Cohort 2

CK5/6 and EGFR immunostains are commonly used as basal markers in TNBC. If EGFR or CK5/6 was positive, then it was considered a basal-like tumor [26]. In Cohort 1, the proportion of basal-like tumors was higher in the TROP2 low group (55.4%) than the high group (44.5%) (*p* = 0.004). In Cohort 2, the proportion of basal-like tumors was higher in the TROP2 low group (30.0%) than in the high group (25.0%), but this was not statistically significant (*p* = 0.675). When the tumor suppressor gene *TP53* is mutated, mutant p53 protein can increase cell proliferation and survival, which contributes to tumor aggressiveness and metastatic potential [27]. Missense mutations can result in high p53 protein expression. This protein is not expressed in deletion mutations, however [28]. Ki-67 immunostain is frequently used as a cellular proliferation cancer marker. A study examining these markers and TROP2 in Cohort 1 and Cohort 2 (Table 3) demonstrated no correlation between them.

**Table 3:** Correlation between expression of TROP2 and phenotype and other proteins (p53 and Ki-67) in two primary TNBC cohorts

Factors	Total (%)	TROP2 expression		*p*-value
**Cohort 1**		Low (%)	High (%)	
Phenotype				.004
Basal	345 (49.3)	170 (55.4)	175 (44.5)	
Non-basal	355 (50.7)	137 (44.6)	218 (55.5)	
p53				.417
< 10	324 (45.3)	148 (47.3)	176 (43.8)	
10–32	64 (9.0)	22 (7.0)	42 (10.4)	
33–65	35 (4.9)	16 (5.1)	19 (4.7)	
≥ 66	292 (40.8)	127 (40.6)	165 (41.0)	
Ki-67 (%)				.711
< 10	39 (6.5)	14 (5.7)	25 (7.0)	
10–19	47 (7.8)	21 (8.6)	26 (7.3)	
≥ 20	514 (85.7)	209 (85.7)	305 (85.7)	
**Cohort 2**		Low (%)	High (%)	
Phenotype				.675
Basal	17 (26.6)	6 (30.0)	11 (25.0)	
Non-basal	47 (73.4)	14 (70.0)	33 (75.0)	
p53				.428
< 10	29 (45.3)	10 (50.0)	19 (43.2)	
10–32	1 (1.6)	0 (0.0)	1 (2.3)	
33–65	3 (4.7)	2 (10.0)	1 (2.3)	
≥ 66	31 (48.4)	8 (40.0)	23 (52.3)	
Ki-67 (%)				.555
< 10	12 (18.8)	5 (25.0)	7 (15.9)	
10–19	2 (3.1)	1 (5.0)	1 (2.3)	
≥ 20	50 (78.1)	14 (70.0)	36 (81.8)	

### TROP2 as a prognostic factor of metastatic TNBC

**Fig. 3:**
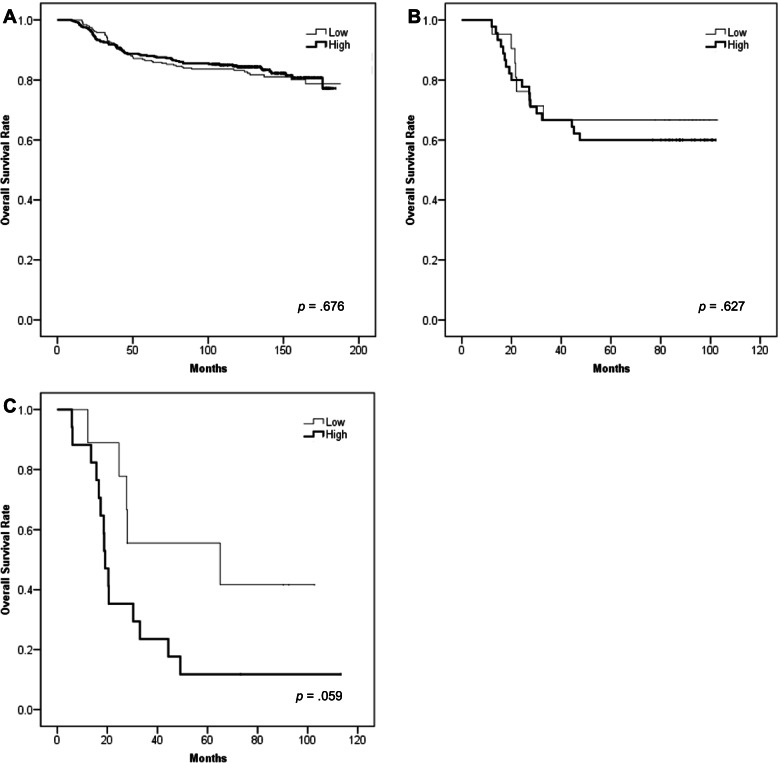
Survival rate of each TNBC cohort. High expression of TROP2 was related to poor prognosis. In the three cohorts, there were no significant differences between TROP2 expression and survival rate. **a** Survival rate of Cohort 1 (*p* = .676, log-rank) **b** Survival rate of Cohort 2 (*p* = .627, log-rank) **c** Survival rate of Cohort 3 (*p* = .059, log-rank)

To identify the prognostic value of TROP2 expression, we used log-rank test and the Kaplan–Meier method to analyze overall survival. The TROP2 high group showed poor OS in Cohort 3, but this was not statistically significant (*p* = 0.059) (Fig. 3).

In the univariate Cox hazards model, TROP2 expression was not a statistically significant prognostic parameter for OS in either the H-score and high vs. low groups in Cohorts 1 and 2. In Cohort 3, TROP2 expression showed statistically significant unfavorable prognosis in H-score (HR = 1.010, 95% confidence interval (CI) = 1.001–1.020, *p* = 0.037) and trended towards an unfavorable prognosis in the high vs. low groups (HR = 2.593, 95% CI = 0.963–7.227, *p* = 0.069) (Table 4). Multivariate analysis including age and TILs demonstrated that TROP2 expression in the H-score group (HR = 1.009, 95% CI = 1.000–1.018, *p* = 0.047) was associated with poor overall survival in Cohort 3.

**Table 4:** Univariate analysis of TNBC prognostic factors among three different cohorts

Factors	Univariate analysis		
	HR	95% CI	*p*-value
Cohort 1			
Age (≥ 50 vs. < 50)	1.132	0.797–1.608	.489
Histologic grade (3 vs. 1 or 2)	0.891	0.604–1.315	.561
Lymph node (positive vs. negative)	2.281	1.609–3.232	< .001*
LVI (positive vs. negative)	3.049	2.152–4.321	< .001*
pT (3 or 4 vs. 1 or 2)	2.392	1.214–4.713	.012*
Stage (2 or 3 vs. 1)	1.740	1.155–2.621	.008*
TILs (%)	0.982	0.974–0.989	< .001*
TROP2 (H-score)	1.001	0.998–1.003	.510
TROP2 (high vs. low)	0.928	0.655–1.316	.676
Cohort 2			
Age (≥ 50 vs. < 50)	0.449	0.134–1.503	.194
Histologic grade (3 vs.1 or 2)	1.361	0.543–3.408	.511
Lymph node (positive vs. negative)	6.774	2.799–16.397	< .001*
LVI (positive vs. negative)	3.294	1.494–7.261	.003*
ypT (3 or 4 vs. 1 or 2)	7.858	3.150–19.600	< .001*
Stage (2 or 3 vs. 1)	7.075	2.416–20.713	< .001*
TILs (%)	0.968	0.938–0.998	.036*
TROP2 (H-score)	1.002	0.995–1.008	.621
TROP2 (high vs. low)	1.241	0.518–2.972	.628
Cohort 3			
Age (≥ 50 vs. < 50)	1.021	0.405–2.577	.964
TILs (%)	0.880	0.769–1.007	.063
TROP2 (H-score)	1.010	1.001–1.020	.037*
TROP2 (high vs. low)	2.593	0.963–7.227	.069

*CI* Confidence interval, *HR* Hazard ratio, *LVI* Lymphovascular invasion, *pT* Pathologic T stage, *TILs* Tumor-infiltrating lymphocytes, *ypT* Post neoadjuvant therapy pathologic T stage; *p < .05

## Discussion

In this study, we investigated TROP2 expression in three different TNBC cohorts. TROP2 expression acquired by H-score showed no difference in the distribution between the three TNBC cohorts. When TROP2 expression in at least 10% of tumor cells is considered as positive, percentage of dominant intensity as moderate to strong expression in the three cohorts was from 80.4% to 89.4%. These results were similar to another study of a metastatic TNBC cohort [21]. In that study, 88% had moderate to strong TROP2 expression. When the expression was divided into positive or negative according to the criteria of 10% of any staining intensity, the positivity was from 92.3% to 98.5%.

There was no difference in clinicopathologic features between the TROP2 high and low groups in primary TNBC tumors (Cohorts 1 and 2). However, in one study of TROP2 membranous expression in invasive ductal breast cancer, the high/low expression of TROP2 was related to histological grade, lymph node metastasis, distant metastasis, TNM staging, cyclin D1, and p53 status [15]. In another study, TROP2 expression was associated with TNBC in breast cancer [24].

TNBC can be divided into basal type and non-basal type subgroups. EGFR and CK5/6 were commonly used as basal markers, but there are no standardized immunohistochemical measurements of EGFR and CK5/6. As a result, EGFR and CK5/6 expression in TNBC ranged from 24–72% and 13–78%, respectively [29]. Although the prevalence of basal markers in TNBC is variable, EGFR had a significant independent prognostic value for disease-free survival in TNBC [30]. CK5/6 is related to a poor prognosis in TNBC [31]. We evaluated the relationship between basal type and TROP2 high and low groups. For tumors positive for EGFR and/or CK5/6, the TROP2 low group had higher ratio of basal type than the TROP2 high group (55.4% in the TROP2 low group and 44.5% in the TROP2 high group in Cohort 1; 30.0% in the TROP2 low group and 25.0% in the TROP2 high group in Cohort 2). While there were no reports on the association of TROP2 and basal markers in breast cancer, one study of lung adenocarcinoma found that the EGFR mutant was in 54% of the TROP2 low group and 46% of the TROP2 high group, but this was not statistically significant (*p* = 0.26) [32].

The *TP53* mutation occurs in approximately 30% of breast cancer cases and 75%-80% of TNBC cases [33]. One study reported that TNBC was more prevalent in cases of high Ki-67 expression (82.5%) than non-TNBC (42.5%) (high expression; ≥ 20%) [34]. We evaluated the p53 and Ki-67 expression in the TROP2 high and low groups. There were no significant differences noted in Cohort 1 and Cohort 2.

TROP2 H-score was a statistically significant unfavorable prognostic marker in the metastatic TNBC cohort (Cohort 3), which had the smallest number of cases. In some studies, the high TROP2 expression group had a shorter survival time than the low group in breast cancer [24, 25]. TROP2 immunohistochemical overexpression has poor overall survival in many solid tumors, like the stomach, nasopharynx, gallbladder, and cervix [35].

Our study results may differ from other studies for several reasons. First, we analyzed only TNBC, but other studies analyzed all breast cancer subtypes. In one study comparing membranous and cytoplasmic TROP2 expression in TNBC and non-TNBC, TNBC had higher TROP2 expression than non-TNBC [24]. More research is needed to confirm the difference between TNBC and non-TNBC. Second, different antibodies have been used in various researches [15, 24, 25]. If a common antibody had been used instead, the results might be more comparable. Finally, we only used excisional specimens to construct the tissue microarray, which might have resulted in a bias.

Although there were some limitations in our study, this is the most comprehensive examination of TROP2 expression in TNBC. We assessed clinicopathologic features and prognosis but also common markers, such as EGFR, CK5/6, p53, and Ki-67. To the best of our knowledge, we compare the TROP2 expression in primary and metastatic TNBCs for the first time. Furthermore, this is the first assessment of TROP2 expression in patients with primary TNBC who received neoadjuvant chemotherapy. However, the results would be better if we analyzed primary and matched metastatic tumors and pre- and post-neoadjuvant chemotherapy tumors together.

A background study on TROP2 expression in a metastatic TNBC cohort receiving the TROP2 ADC reported that most tumors (88%) were moderately or strongly positive for TROP2 expression. This is a major reason that TROP2 expression is not a condition for using the drug [21]. In our study, 84.6% was a moderate-to-strong positive for TROP2 in the metastatic cohort. We conducted the study on only metastatic specimens in Cohort 3 and found that high TROP2 expression had a poor prognosis. In a previous study, a longer progression-free survival was observed in the moderate to strong TROP2 group, so the patients with high TROP2 expression can benefit from the drug. To accurately establish drug indications and usage guidelines, further research should clarify if patients with low TROP2 expression can benefit from the drug.

## Conclusions

We showed that TROP2 expression of TNBC is similar regardless of neoadjuvant treatment or primary tumor/metastasis. TROP2 expression was revealed as a poor prognostic factor in metastatic TNBC, therefore some patients with high TROP2 expression may benefit from ADC for TROP2. Further evaluation of the predictive value of TROP2 expression and establishment of indication for targeted therapy should be performed.

## Supplementary Information

**Additional file 1: Supplementary Table 1.** Clinicopathologic features in three different cohorts. **Supplementary Figure 1.** Representative TROP2 expression in normal skin (positive control) and normal cerebral cortex (negative control). Original magnification x400. a skin (TROP2 antibody), b cerebral cortex (TROP2 antibody), c skin (rabbit IgG), d cerebral cortex (rabbit IgG).

## Data Availability

The datasets used and/or analyzed during the current study are available from the corresponding author on reasonable request.

## Abbreviations

TROP2: Trophoblast cell-surface antigen 2
TNBC: Triple-negative breast cancer
ER: Estrogen receptor
PR: Progesterone receptor
HER2: Human epidermal growth factor receptor 2
ADC: Antibody–drug conjugate
LVI: Lymphovascular invasion
TILs: Tumor-infiltrating lymphocytes
EGFR: Epidermal growth factor receptor
CK5/6: Cytokeratin 5/6
IBC-NST: Invasive breast carcinoma of no special type
