# Supplementary material for: Trophoblast cell-surface antigen 2 (TROP2) expression in triple-negative breast cancer
Source: BMC Cancer. 2022 Sep 24;22:1014. doi: 10.1186/s12885-022-10076-7 (PMC9509625; doi:10.1186/s12885-022-10076-7)
Supplement: Supplementary file 1 — Additional file 1: Supplementary Table 1. Clinicopathologic features in three different cohorts. Supplementary Figure 1. Representative TROP2 expression in normal skin (positive control) and normal cerebral cortex (negative control). Original magnification x400. a skin (TROP2 antibody), b cerebral cortex (TROP2 antibody), c skin (rabbit IgG), d cerebral cortex (rabbit IgG). [file 12885_2022_10076_MOESM1_ESM.doc]

**Supplementary Table 1.** Clinicopathologic features in three different cohorts

| Factors | Cohort 1 (%) | Cohort 2 (%) | Cohort 3 (%) |
| --- | --- | --- | --- |
| Age (yr) |  |  |  |
| < 50 | 426 (59.6) | 52 (78.8) | 18 (69.2) |
| ≥ 50 | 289 (40.4) | 14 (21.2) | 8 (30.8) |
| Nuclear grade |  |  |  |
| 1 | 0 | 0 | 0 |
| 2 | 184 (25.7) | 16 (24.2) | 0 |
| 3 | 531 (74.3) | 50 (75.8) | 26 (100) |
| Histologic grade |  |  |  |
| 1 | 1 (0.1) | 0 | 0 |
| 2 | 177 (24.8) | 19 (28.8) | 0 |
| 3 | 537 (75.1) | 47 (71.2) | 26 (100) |
| Histologic type |  |  |  |
| IBC-NST | 596 (83.4) | 61 (92.4) | 26 (100) |
| Metaplastic | 66 (9.2) | 5 (7.6) | 0 |
| Apocrine | 26 (3.6) | 0 | 0 |
| Micropapillary | 21 (2.9) | 0 | 0 |
| Mucinous | 1 (0.1) | 0 | 0 |
| Adenoid cystic | 4 (0.6) | 0 | 0 |
| Lobular | 1 (0.1) | 0 | 0 |
| pT/ypT |  |  |  |
| 1 | 305 (42.7) | 1 (1.5) |  |
| 2 | 384 (53.7) | 42 (63.6) |  |
| 3 | 25 (3.5) | 15 (22.7) |  |
| 4 | 1 (0.1) | 8 (12.1) |  |
| pN/ypN |  |  |  |
| 0 | 467 (65.3) | 40 (60.6) |  |
| 1 | 152 (21.3) | 13 (19.7) |  |
| 2 | 53 (7.4) | 7 (10.6) |  |
| 3 | 43 (6.0) | 6 (9.1) |  |
| LVI |  |  |  |
| Negative | 537 (75.1) | 46 (69.7) |  |
| Positive | 178 (24.9) | 20 (30.3) |  |
| Stage |  |  |  |
| 1 | 241 (33.7) | 31 (47.0) |  |
| 2 | 372 (52.0) | 18 (27.3) |  |
| 3 | 102 (14.3) | 17 (25.8) |  |
| TILs (%) |  |  |  |
| <2 | 30 (4.2) | 20 (30.3) | 7 (26.9) |
| 2-9 | 139 (19.4) | 15 (22.7) | 13 (50.0) |
| 10-59 | 320 (44.8) | 26 (39.4) | 6 (23.1) |
| 60-100 | 226 (31.6) | 5 (7.6) | 0 |
|  |  |  |  |

IBC-NST, invasive breast carcinoma of no special type; pT, pathologic T stage; ypT, post neoadjuvant therapy pathologic T stage; pN, pathologic N stage; ypN, post neoadjuvant therapy pathologic N stage; LVI, lymphovascular invasion; TILs, tumor-infiltrating lymphocytes;


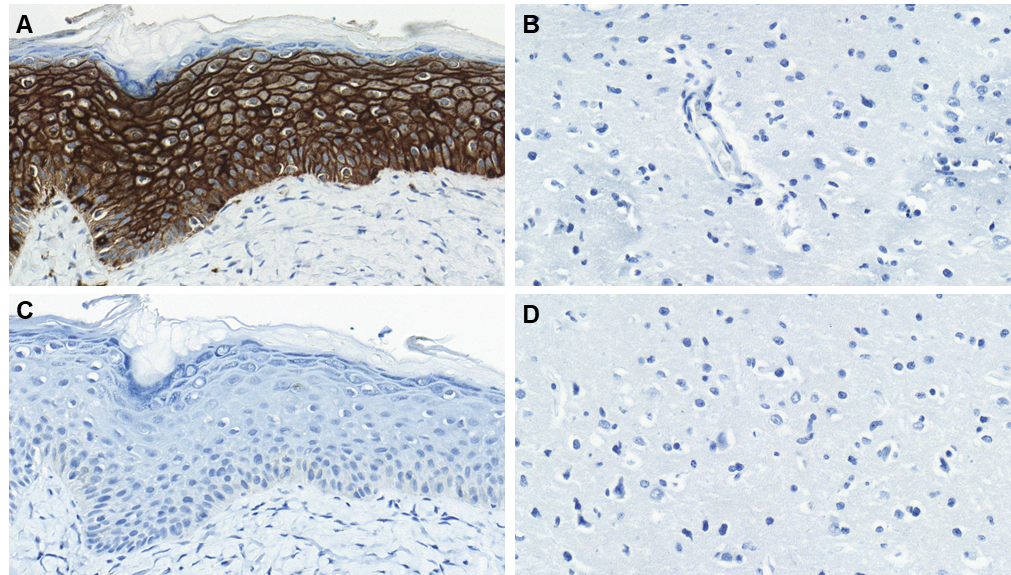


**Supplementary Fig. 1** Representative TROP2 expression in normal skin (positive control) and normal cerebral cortex (negative control). Original magnification x400. **a** skin (TROP2 antibody), **b** cerebral cortex (TROP2 antibody), **c** skin (rabbit IgG), **d** cerebral cortex (rabbit IgG)
